# Neuroassessment in Sports: An Integrative Approach for Performance and Potential Evaluation in Athletes

**DOI:** 10.3389/fpsyg.2022.747852

**Published:** 2022-04-14

**Authors:** Davide Crivelli, Michela Balconi

**Affiliations:** ^1^International Research Center for Cognitive Applied Neuroscience (IrcCAN), Catholic University of the Sacred Heart, Milan, Italy; ^2^Research Unit in Affective and Social Neuroscience, Department of Psychology, Catholic University of the Sacred Heart, Milan, Italy

**Keywords:** neuroassessment, sport, neurocognitive efficiency, attention regulation, focusing, self-regulation, neurocognitive fitness, peak performance

## Performance Determinants: Physical, Psychological, and Neurocognitive Fitness

Peak performance—as the traditional Olympic motto “*Citius, Altius, Fortius*” (“*Faster, Higher, Stronger*”) points out—is a primary goal for athletes, but it also can be deemed—as posited by Pierre de Coubertin, promoter of the modern Olympic Games—as one of the primary personal development goals which drives peoples every day and professional lives. This makes research in sports a critical window into the advancement of our understanding on how to foster, evaluate, and empower human performance.

The profiling of athletes' psychophysical traits is, in particular, a crucial step in planning, implementing, and monitoring their training schedule and, therefore, receives particular attention by the athletes themselves, their coaches, and sports clubs. Performance data, if generated by appropriately designed assessments, may provide relevant and reliable information to evaluate the progress of athletes over time and/or rank them against their peers (Robertson et al., 2014). Further, such data also help to profile the athlete's strengths/weaknesses, to monitor progress, to adapt training protocols to the athlete's needs, and to identify talent or to predict the athlete's potential (e.g., Morris-Binelli et al., 2021). Yet, the topics of assessment and, specifically, of its implementation in practice are still quite controversial. Indeed, despite the relationship between athletic skill efficiency and success in sports, literature reviewing psychometric properties of assessment methods (e.g., validity, reliability, and predictive value) is still scant (Currell and Jeukendrup, 2008; Robertson et al., 2014; Piggott et al., 2020). Furthermore, as underlined by Robertson et al. (2014), outdated methodology and undefined measurement properties actually limit the usefulness of many performance and skill measures. This is particularly true for their actual implementation in routine professional practice - even though they might be widely used in research - with remarkable implications for assessing the effect of coaching, fatigue, focus of attention, and pre-skill execution routine on participant performance (Mccann et al., 2001; Russell and Kingsley, 2011; Russell et al., 2011; Mckay and Wulf, 2012).

Again, despite the fact that the contribution of psychological factors to individual sport-related experience and that the role of psychological/cognitive load in modulating performance outcomes are widely recognized (Mellalieu et al., 2021), the assessment procedures to evaluate athletes' current performance and their potential often move those factors to the background. The vast majority of empirical work, indeed, focuses primarily on the physical determinants of performance and basic physiological measures. The paucity of suitable tools and standardized procedures for the assessment of psychological and cognitive correlates/determinants of sport performance (e.g., self-regulation skills, set-shifting efficiency, and cognitive control) and of specific training of technical staff in their use has likely contributed to the definition of a theoretical and methodological framework almost uniquely centered on physical fitness.

Namely, *physical fitness* (PhyF) is a well-established construct, whose core components include muscular strength, endurance, and range of motion/joint flexibility (Jeffreys and Moody, 2016). Transdisciplinary as well as sport-specific metrics exist for each of those core elements of PhyF. Similarly, specific training protocols have been developed to enhance strength, endurance and/or flexibility (e.g., conditioning training, high-intensity interval training, and stretching routines; Jeffreys and Moody, 2016). Nonetheless, the interdependence of physical, affective, and cognitive factors in contributing to athletic performance is clearly pointed out by a solid evidence base (Piggott et al., 2020). Across the years, more and more athletes and sport professionals have begun to look at mental factors less as corollary elements for performance assessment (Habay et al., 2021; Mellalieu et al., 2021), and mental training practices are becoming less exclusive.

The construct of *psychological fitness* (PsyF; Heaps, 1978) was then introduced even in sport science and practice to identify various psychological traits—including character strengths, personality traits, personal resources, and resilience against burnout or mental disorders (Heaps, 1978; Cornum et al., 2011; Wesemann et al., 2018)—that promote wellbeing, foster personal development, and sustain performance. Cognitive flexibility and executive functions are, as an example, known as a broad-spectrum protective factor in preventing mental illness (Crivelli and Balconi, 2021) as well as in sustaining personal development (Keyes, 2007; Robinson et al., 2015). Again, empowerment protocols focusing on PsyF have been devised and applied for performance improvement and enhancement of training outcomes even in the army and in sports (Birrer and Morgan, 2010; Boga, 2017). Individual and team ability to manage the psychological load in complex challenging situations is widely deemed as conferring critical performance advantages (Aidman, 2020; Mellalieu et al., 2021).

Yet, we suggest that at least a third level should be considered in defining a comprehensive account for assessment and monitoring of athletes' performance and potential, which could be defined *neurocognitive fitness* (NCF). The construct of NCF builds on the cognitive fitness framework (Aidman, 2020), a working hypothesis intended to integrate available evidences on the contribution of cognitive skills training to performance enhancement, while extending that seminal concept by using a psychophysiological and neurofunctional perspective. Therefore, NCF could be understood as the degree of efficacy and efficiency in exploiting available neural and cognitive resources to complete a task based on the challenge level that the task itself and the environment impose, in order to exert an optimal level of performance. Core components of NCF, as we define it, include: self-awareness, as a primary precursor of intentional goal-directed behavior; self-regulation, as the background for stress management and self-control; and executive control, as the intersection of attention regulation, cognitive flexibility, cognitive workload management, and inhibition skills. As underlined by Nakata et al. (2010), to reach optimal performance and perform skilled movements in real-life sport situations, an athlete needs to be able to flexibly and efficiently adapt movements constituting the athletic gesture based on the perception of environmental information, discrimination of relevant stimuli, rapid decision-making processes, integration of afferent signals, and anticipatory action preparation. Set-shifting, behavioral inhibition, focusing and attention regulation mechanisms are all the same crucial to reach and maintain high-performance levels.

Notably, neurocognitive empowerment protocols based on the combined use of cognitive techniques (e.g., focused attention and self-awareness practices) and neuromodulation devices (e.g., wearable neurofeedback) have already been shown to promote the efficiency of attention regulation and executive control, besides greater stress management skills, even in applied contexts, such as at the workplace and in sports (Balconi et al., 2019a,b; Crivelli et al., 2019a,b). Such protocols, by combining neurotechnologies able to promote greater control on neural resources and cognitive-behavioral practices supporting self-enhancement, represent a valuable example of training programmes implementing NCF in real-life contexts. Such effective integration between psychological, cognitive and neurofunctional levels is likely not that mature in the application field concerning performance profiling and assessment, with the majority of solutions implemented in practice still mainly based on behavioral, psychological, and (limitedly) neuropsychological testing.

## From Assessment to Neuroassessment

While the investigation of behavioral, psychological and cognitive determinants of personal performance in profiling an athlete's current strengths/weaknesses and potential should be deemed, as above noted, highly relevant, we here intend to point out the potential of an actual integration of those measures with objective neurofunctional and autonomic markers, according to a neuro-behavioral perspective and capitalizing on embodied and perception-action theoretical frameworks (Aglioti et al., 2008; Müller and Abernethy, 2012; Wright et al., 2013). The human brain indeed shows, together with our bodies, the remarkable ability to learn and capitalize from experience to reach mastery and excellence in specialized skills, and that is particularly important when focusing on the investigation of peak performance and highly specialized activities, such as in sport practice. Namely, by accurately qualifying and quantifying the cascade of physiological events that mark cognitive-motor learning, attention focusing, and self-regulation, as well as planning, programming, and executing athletic gestures, and by linking them to behavior and psychological processes, it is possible to infer how our brain functions during challenging tasks and contexts. Such an evidence base helps identifying factors promoting optimal performance for the examined athlete (Perrey, 2008; Thompson et al., 2008; Piggott et al., 2020).

A vast literature coming from basic neuroscience research already provides a remarkable evidence base for understanding how athletes' brains support them during sport and exercise activity and also when, where and to what extent an athlete brain is different from the one of a common person (Nakata et al., 2010; Li and Smith, 2021). Integrating psychometric, behavioral, and observational assessment tools with neuroscientific devices able to capture covert physiological markers of the efficiency of processes supporting executive control and attention regulation also represents a valuable advantage in the quest for novel, objective, and actually predictive tools for assessment of performance in sport contexts, as is beginning to happen even in other applied contexts (Balconi et al., 2020).

By facilitating insights into sensory, motor, and cognitive processes contributing to the preparation, execution and imagination of the athletic gesture, as well as to the orientation of attention resources on competition, both wearable and lab-based neuroscientific devices (among which, in particular, electroencephalography - EEG, autonomic indices, and—more recently—functional Near Infrared Spectroscopy—fNIRS) may also help in shedding light on specific aspects of sport activity and its neurofunctional characteristics (Crivelli and Balconi, 2017; Balconi and Crivelli, 2019)—such as, for instance, modulations of cognitive effort in pre-competition phases, training-induced enhancement of stimuli detection, resistance to mental/physical fatigue and its relation to performance. Integrating such devices in established assessment procedures could foster the adoption of a multifaceted analytical approach that properly include the investigation of neurocognitive fitness, together with psychological and physical ones. It has to be noted that the topic of feasibility of neurofunctional-psychophysiological assessment in ecological settings is actually still a hot topic in sport and exercise science. Yet methodological debate on such a topic has highlighted interesting developments during recent years and recommendations concerning subject preparation, sensor placement, environmental controls, use of portable recording devices, and signal processing have been proposed to improve quality and informativity of physiological data captured even outside of the lab and during actual exercise/sport activity (for reviews, see Perrey, 2008; Thompson et al., 2008; Park et al., 2015; Cheron et al., 2016; Balconi and Crivelli, 2019).

We therefore propose that the time has come for a more systematic implementation of the perspective change from assessment to *neuroassessment*. Neuroassessment can be defined as a standardized procedure to qualify and quantify the level of physical, psychological and neurocognitive fitness of an athlete via a combination of self-report, behavioral, autonomic, and neurofunctional metrics. Those metrics should be able to capture different and complementary facets of performance during standardized as well as ecologically-valid sport-specific tasks. Notably, the systematic progress in bioengineering and medical technologies allows, to date, to bring neuroscientific tools for biometric measurements even on the field, thus making it possible to collect valuable data during field based sessions of physical and mental training.

Also, such rich sets of data would not only result in a gain in insight for the purposes of athletes profiling and planned development, but will also help building stronger and more complete theoretical frameworks to classify athletes self-awareness, self-regulation, and higher cognition (e.g., attention regulation, inhibitory mechanisms, and information-processing and focusing) skills and their physiological signatures, in keeping with neuroscientific models of such skills.

## Profiling Attention Regulation Skills: an Applied Example

By taking attention regulation as an example, we will now briefly introduce a neuroassessment protocol devised to improve evaluation of attention focusing in sports.

The ability to focus attention on target stimuli relevant to the sport context and specific discipline while inhibiting irrelevant information notwithstanding their perceptual or affective salience is considered a key aspect of optimal performance and a valuable transdisciplinary trait in athletes (Memmert, 2009). Yet, notwithstanding the acknowledged relevance of those abilities, profiling athletes on their primary attention regulation and control skills is still commonly based on psychometric testing drawing from the classical theory of attentional styles by Nideffer (1976). Also, psychometric testing, as well as purely behavioral measures, presents a few limitations when used by themselves to try “opening the black box” since, for example, they are susceptible to desirability biases and limited self-reflection abilities. Moreover, they could hardly parse out the contribution of strategic top-down vs. instinctual bottom-up processes to attentional performance. Such fine-grained analysis could be more easily obtained by using neurofunctional techniques.

The proposed neuroassessment protocol, in particular, integrates: self-report evaluation of the ability to regulate attention in sport/competitive contexts (self-awareness component), computerized and neuropsychological testing of the efficiency of attention regulation (behavioral performance component), as well as autonomic (EDA, HR, HRV) and electrophysiological markers of neurocognitive efficiency (task-related alpha-beta modulations as markers of cognitive workload (Janelle and Hatfield, 2008; Thompson et al., 2008), N2 and P3 event-related potentials as markers of attention regulation and stimuli detection (Nakata et al., 2010; Balconi and Crivelli, 2019), Event-Related Negativity as a marker of monitoring processes (Themanson et al., 2008; Masaki et al., 2017) during challenging ecological tasks implementing salient stimuli evoking sport-specific contexts (psychophysiological and neurofunctional components). Namely, one of the tasks we have developed and tested is an adapted, unpublished, version of a cueing task with multiple target positions and ecological stimuli specific to different sport disciplines (e.g., in fighting sports, fist and kick strikes as target stimuli and guard of an opponent fighter as endogenous cue). Given its characteristics, this exemplifying protocol can be used to profile athletes and inform the design of personalized empowerment programs.

Preliminary validation data from the above-mentioned project highlighted internally consistent profiles across the multi-dimensional metrics of attention regulation and executive control performance, hinting at the potential of the protocol for in-depth assessment of athletes' main characteristics and at the complementarity of chosen performance measures.

## Conclusion

The conceptualization of a multifactorial construct, as suggested by Atkinson (2002), facilitates the definition of its measurable components. Here we have introduced a multifaceted reference model for the definition of performance in sports by pairing the more established constructs of PhyF and PsyF with the construct of NCF. Such a model might provide the framework for a leaner perspective change from traditional observational or physical assessment procedures to neuroassessment, which we identify with the actual integration of both subjective (self-report, observational) and objective (behavioral, physiological) measures to sketch the profile of athletes' neurocognitive efficiency.

## Author Contributions

DC and MB contributed to conception of the present work. DC wrote the first draft of the manuscript and MB revised it. All authors have read and approved the submitted version.

## Conflict of Interest

The authors declare that the research was conducted in the absence of any commercial or financial relationships that could be construed as a potential conflict of interest.

## Publisher's Note

All claims expressed in this article are solely those of the authors and do not necessarily represent those of their affiliated organizations, or those of the publisher, the editors and the reviewers. Any product that may be evaluated in this article, or claim that may be made by its manufacturer, is not guaranteed or endorsed by the publisher.

## References

[B1] Aglioti S. M. Cesari P. Romani M. Urgesi C. (2008). Action anticipation and motor resonance in elite basketball players. Nat. Neurosci. 11, 1109–1116. 10.1038/nn.2182 19160510

[B2] Aidman E. (2020). Cognitive fitness framework: towards assessing, training and augmenting individual-difference factors underpinning high-performance cognition. Front. Hum. Neurosci. 13, 1–9. 10.3389/fnhum.2019.00466 32009919 PMC6971199

[B3] Atkinson G. (2002). Sport performance: variable or construct? J. Sports Sci. 20, 291–292. 10.1080/026404102753576053 12003273

[B4] Balconi M. Angioletti L. Crivelli D. (2020). Neuro-empowerment of executive functions in the workplace: the reason why. Front. Psychol. 11:1519. 10.3389/fpsyg.2020.01519 32848981 PMC7411509

[B5] Balconi M. Crivelli D. (2019). “Fundamentals of electroencephalography and optical imaging for sport and exercise science. From the laboratory to on-the-playing-field acquired evidence,” in Handbook of Sport Neuroscience and Psychophysiology, eds R. A. Carlstedt and M. Balconi (New York, NY: Routledge), 40–69. 10.4324/9781315723693-3

[B6] Balconi M. Crivelli D. Angioletti L. (2019a). Efficacy of a neurofeedback training on attention and driving performance: physiological and behavioral measures. Front. Neurosci. 13:996. 10.3389/fnins.2019.00996 31619958 PMC6760023

[B7] Balconi M. Crivelli D. Fronda G. Venturella I. (2019b). “Neuro-rehabilitation and neuro-empowerment by wearable devices. Applications to well-being and stress management,” in Converging Clinical and Engineering Research on Neurorehabilitation III Biosystems & Biorobotics, eds L. Masia, S. Micera, M. Akay, and J. L. Pons (Cham: Springer International Publishing), 963–966. 10.1007/978-3-030-01845-0_193

[B8] Birrer D. Morgan G. (2010). Psychological skills training as a way to enhance an athlete's performance in high-intensity sports. Scand. J. Med. Sci. Sports 20, 78–87. 10.1111/j.1600-0838.2010.01188.x 20840565

[B9] Boga D. (2017). “Training mental fitness,” in Human Dimension, ed Australian Army Headquarters (Puckapunyal: Centre for Army Lessons, Army Knowledge Group), 18–27.

[B10] Cheron G. Petit G. Cheron J. Leroy A. Cebolla A. Cevallos C.. (2016). Brain oscillations in sport: toward EEG biomarkers of performance. Front. Psychol. 7:246. 10.3389/fpsyg.2016.00246 26955362 PMC4768321

[B11] Cornum R. Matthews M. D. Seligman M. E. P. (2011). Comprehensive soldier fitness: building resilience in a challenging institutional context. Am. Psychol. 66, 4–9. 10.1037/a0021420 21219042

[B12] Crivelli D. Balconi M. (2017). Event-related electromagnetic responses. Ref. Modul. Neurosci. Biobehav. Psychol. 4, 1–27. 10.1016/B978-0-12-809324-5.03053-4

[B13] Crivelli D. Balconi M. (2021). “Psychopathology of EFs,” in Advances in Substance and Behavioral Addiction - The Role of Executive Functions, eds M. Balconi and S. Campanella (Cham: Springer), 2. 10.1007/978-3-030-82408-2_2

[B14] Crivelli D. Fronda G. Balconi M. (2019a). Neurocognitive enhancement effects of combined mindfulness–neurofeedback training in sport. Neuroscience 412, 83–93. 10.1016/j.neuroscience.2019.05.066 31195055

[B15] Crivelli D. Fronda G. Venturella I. Balconi M. (2019b). Stress and neurocognitive efficiency in managerial contexts: a study on technology-mediatedmindfulness practice. Int. J. Work. Heal. Manag. 12, 42–56. 10.1108/IJWHM-07-2018-0095

[B16] Currell K. Jeukendrup A. (2008). Validity, reliability and sensitivity of measures of sporting performance. Sport. Med. 38, 297–316. 10.2165/00007256-200838040-00003 18348590

[B17] Habay J. Van Cutsem J. Verschueren J. De Bock S. Proost M. De Wachter J.. (2021). Mental fatigue and sport-specific psychomotor performance: a systematic review. Sport. Med. 51, 1527–1548. 10.1007/s40279-021-01429-6 33710524

[B18] Heaps R. A. (1978). Relating physical and psychological fitness: a psychological point of view. J. Sports Med. Phys. Fitness 18, 399–408. 745389

[B19] Janelle C. M. Hatfield B. D. (2008). Visual attention and brain processes that underlie expert performance: implications for sport and military psychology. Mil. Psychol. 20, 39–69. 10.1080/08995600701804798

[B20] Jeffreys I. Moody J. (2016). Strength and Conditioning for Sports Performance. London: Routledge. 10.4324/9780203852286

[B21] Keyes C. L. M. (2007). Promoting and protecting mental health as flourishing: a complementary strategy for improving national mental health. Am. Psychol. 62, 95–108. 10.1037/0003-066X.62.2.95 17324035

[B22] Li L. Smith D. M. (2021). Neural efficiency in athletes: a systematic review. Front. Behav. Neurosci. 15:698555. 10.3389/fnbeh.2021.698555 34421553 PMC8374331

[B23] Masaki H. Maruo Y. Meyer A. Hajcak G. (2017). Neural correlates of choking under pressure: athletes high in sports anxiety monitor errors more when performance is being evaluated. Dev. Neuropsychol. 42, 104–112. 10.1080/87565641.2016.1274314 28452597

[B24] Mccann P. Lavallee D. Lavallee R. (2001). The effect of pre-shot routines on golf wedge shot performance. Eur. J. Sport Sci. 1, 1–10. 10.1080/17461390100071503

[B25] Mckay B. Wulf G. (2012). A distal external focus enhances novice dart throwing performance. Int. J. Sport Exerc. Psychol. 10, 149–156. 10.1080/1612197X.2012.682356

[B26] Mellalieu S. Jones C. Wagstaff C. Kemp S. Cross M. J. (2021). Measuring psychological load in sport. Int. J. Sports Med. 42, 782–788. 10.1055/a-1446-9642 33862638

[B27] Memmert D. (2009). Pay attention! A review of visual attentional expertise in sport. Int. Rev. Sport Exerc. Psychol. 2, 119–138. 10.1080/17509840802641372

[B28] Morris-Binelli K. Müller S. van Rens F. E. C. A. Harbaugh A. G. Rosalie S. M. (2021). Individual differences in performance and learning of visual anticipation in expert field hockey goalkeepers. Psychol. Sport Exerc. 52:101829. 10.1016/j.psychsport.2020.101829 34897232

[B29] Müller S. Abernethy B. (2012). Expert anticipatory skill in striking sports. Res. Q. Exerc. Sport 83, 175–187. 10.1080/02701367.2012.10599848 22808703

[B30] Nakata H. Yoshie M. Miura A. Kudo K. (2010). Characteristics of the athletes' brain: evidence from neurophysiology and neuroimaging. Brain Res. Rev. 62, 197–211. 10.1016/j.brainresrev.2009.11.006 19944119

[B31] Nideffer R. M. (1976). Test of attentional and interpersonal style. J. Pers. Soc. Psychol. 34, 394–404. 10.1037/0022-3514.34.3.394

[B32] Park J. L. Fairweather M. M. Donaldson D. I. (2015). Making the case for mobile cognition: EEG and sports performance. Neurosci. Biobehav. Rev. 52, 117–130. 10.1016/j.neubiorev.2015.02.014 25735956

[B33] Perrey S. (2008). Non-invasive NIR spectroscopy of human brain function during exercise. Methods 45, 289–299. 10.1016/j.ymeth.2008.04.005 18539160

[B34] Piggott B. Müller S. Chivers P. Cripps A. Hoyne G. (2020). Interdisciplinary sport research can better predict competition performance, identify individual differences, and quantify task representation. Front. Sport. Act. Living 2:14. 10.3389/fspor.2020.00014 33345009 PMC7739773

[B35] Robertson S. J. Burnett A. F. Cochrane J. (2014). Tests examining skill outcomes in sport: a systematic review of measurement properties and feasibility. Sport. Med. 44, 501–518. 10.1007/s40279-013-0131-0 24293244

[B36] Robinson P. Oades L. G. Caputi P. (2015). Conceptualising and measuring mental fitness: a Delphi study. Int. J. Wellbeing 5, 53–73. 10.5502/ijw.v5i1.4

[B37] Russell M. Benton D. Kingsley M. (2011). The effects of fatigue on soccer skills performed during a soccer match simulation. Int. J. Sports Physiol. Perform. 6, 221–233. 10.1123/ijspp.6.2.221 21725107

[B38] Russell M. Kingsley M. (2011). Influence of exercise on skill proficiency in soccer. Sport. Med. 41, 523–539. 10.2165/11589130-000000000-00000 21688867

[B39] Themanson J. R. Pontifex M. B. Hillman C. H. (2008). Fitness and action monitoring: evidence for improved cognitive flexibility in young adults. Neuroscience 157, 319–328. 10.1016/j.neuroscience.2008.09.014 18845227 PMC2657808

[B40] Thompson T. Steffert T. Ros T. Leach J. Gruzelier J. (2008). EEG applications for sport and performance. Methods 45, 279–288. 10.1016/j.ymeth.2008.07.006 18682293

[B41] Wesemann U. Willmund G. D. Ungerer J. Kreim G. Zimmermann P. L. Bühler A.. (2018). Assessing psychological fitness in the military – development of an effective and economic screening instrument. Mil. Med. 183, e261–e269. 10.1093/milmed/usy021 29596663

[B42] Wright M. J. Bishop D. T. Jackson R. C. Abernethy B. (2013). Brain regions concerned with the identification of deceptive soccer moves by higher-skilled and lower-skilled players. Front. Hum. Neurosci. 7:851. 10.3389/fnhum.2013.00851 24381549 PMC3865769

